# Carbon Microparticles from Organosolv Lignin as Filler for Conducting Poly(Lactic Acid)

**DOI:** 10.3390/polym8060205

**Published:** 2016-05-26

**Authors:** Janea Köhnke, Christian Fürst, Christoph Unterweger, Harald Rennhofer, Helga C. Lichtenegger, Jozef Keckes, Gerhard Emsenhuber, Arunjunai raj Mahendran, Falk Liebner, Wolfgang Gindl-Altmutter

**Affiliations:** 1 BOKU-University of Natural Resources and Life Science Vienna, Konrad Lorenz Strasse 24, A-3430 Tulln, Austria; gerhard.emsenhuber@boku.ac.at (G.E.); falk.liebner@boku.ac.at (F.L.); wolfgang.gindl-altmutter@boku.ac.at (W.G.-A.); 2 Kompetenzzentrum Holz GmbH, Altenbergerstrasse 89, A-4020 Linz, Austria; c.fuerst@kplus-wood.at (C.F.); c.untergweger@kplus-wood.at (C.U.); a.mahendran@kplus-wood.at (A.R.M.); 3 BOKU-University of Natural Resources and Life Science Vienna, Peter Jordan Strasse 82, A-1190 Vienna, Austria; harald.rennhofer@boku.ac.at (H.R.); helga.lichtenegger@boku.ac.at (H.C.L.); 4 Department Materials Physics, University of Leoben, Jahnstrasse 12, A-8700 Leoben, Austria; jozef.keckes@unileoben.ac.at

**Keywords:** lignin, carbon, carbonisation, electric conductivity, SAXS, filled polymers, tensile testing

## Abstract

Carbon microparticles were produced from organosolv lignin at 2000 °C under argon atmosphere following oxidative thermostabilisation at 250 °C. Scanning electron microscopy, X-ray diffraction, small-angle X-ray scattering, and electro-conductivity measurements revealed that the obtained particles were electrically conductive and were composed of large graphitic domains. Poly(lactic acid) filled with various amounts of lignin-derived microparticles showed higher tensile stiffness increasing with particle load, whereas strength and extensibility decreased. Electric conductivity was measured at filler loads equal to and greater than 25% *w*/*w*.

## 1. Introduction

Lignin is the second most abundant natural polymer. It is an amorphous, thermoplastic, three-dimensional, cross-linked and aromatic component of the cell wall, that consists of substituted phenylpropane units, whose pattern largely depends on the plant species. Formed by random radical dehydropolymerisation of the three main aromatic precursor compounds coumaryl, coniferyl and sinapyl alcohol, lignin has a great diversity of bonds and functional groups [1]. ß-O-4 bonds can account for up to 70 percent of all linkages between lignols, but other bond types play a crucial role with regard to lignin properties as well, such as ether and C–C bonds as found in ß-5 and 5-5′ structural units. This applies for the functional groups of lignin as well which comprise in particular methoxyl, carbonyl, carboxyl and hydroxyl groups [2].

Lignin is an essential component of lignocellulosic plant tissues where it acts as a connecting, stiffening matrix between cells, increases compression resistance, imparts hydrophobicity to the cell wall, and provides protection against microorganisms [3]. Lignocellulosic materials such as wood contain between about 15 and 40 percent lignin, which can be extracted to a large extent during wood pulping. Taking into consideration global pulp production, about 70 million tons of lignin are produced as a side product every year, dominated by kraft lignin, soda lignin and the various types of lignosulfonates [4]. Whilst the majority of lignin is still used as fuel despite its comparatively low heating value, only 1.7 percent of this valuable natural resource finds application in materials.

Recent studies have confirmed renewed interest in using lignin as feedstock for the production of materials such as dispersants, binders or bulking agents [4]. A particular focus is currently on lignin-derived carbon fibres [5,6,7,8], which are hoped to become a low-cost alternative to high-quality carbon fibres derived from polyacrylonitrile. The application envisioned for lignin-derived carbon fibres is in lighter and hence fuel saving transportation devices.

Besides carbon fibre, carbon black has become a material that is of great technological and industrial importance because of its unique mechanical, chemical and electrical properties [9,10,11]. On the one hand, carbon black may reduce the overall material costs as low-cost filler in polymers, and on the other hand carbon black as filler can significantly improve the overall properties of plastics and elastomers, especially electric conductivity [12,13]. Carbon black is obtained through incomplete combustion or thermal degradation of fossil resources in absence of oxygen. According to the International Carbon Black Association (http://www.carbon-black.org), more than 8 million metric tons of carbon black were produced worldwide in 2014, almost exclusively from fossil resources. Therefore, a viable bio-based alternative to fossil-derived carbon black is of great interest. It is proposed that lignin-derived carbon microparticles may represent such an alternative. In the present study we focus on organosolv lignin as a potential raw material for carbonisation. The organosolv pulping process, which is based on the lignin solubilisation ability of (acidic) aqueous alcohols at elevated temperature and pressure, is considered to have great potential for lignocellulose biorefinery as demonstrated by the pilot scale plant Leuna [14], even though commercial implementation has not happened yet. Numerous studies [15,16,17,18] have confirmed that organosolv lignins have superior quality because of their high purity, low molecular weight and narrow size distribution as well as their low sulfur and salt contents. In view of these advantageous properties, the feasibility of producing carbon microparticles from organosolv lignin will be evaluated together with selected properties of this novel material.

## 2. Materials and Methods

### 2.1. Materials

Organosolv lignin (Product number CP8068-03-9-BULK) was purchased from Chemical Point UG (Deisenhofen, Germany) (Element contents: C: 61.7%, H: 5.3%, N: 0.9%, S: 0%, O: 28.3%, rest: 3.81%. Chloroform (Purity: ≥99.5% was obtained from Sigma-Aldrich (Vienna, Austria) and poly(lactic acid) grade 2003D was purchased from Natureworks LLC (Minnetonka Boulevard, Minnetonka, MN, USA).

### 2.2. Lignin Carbonisation

Prior to carbonisation, lignin was thermostabilised to prevent particles from fusing during pyrolysis and graphitisation. For this purpose, organosolv lignin was evenly distributed in a crucible and heated at a rate of 0.01 min^−1^ to 250 °C in ambient atmosphere using a drying oven. Carbonisation was conducted in a GERO HTK8 oven (volume 6 L) using an argon gas flow of 150 L·h^−1^. The oven was set to a heating rate of 1 °C·min^−1^ up to 500 °C (1 h holding step), 5 °C·min^−1^ up to 900 °C (1 h holding step) and finally 5 °C·min^−1^ up to 2000 °C (1 h holding step).

### 2.3. Characterisation of Lignin and Carbon Particles

For scanning electron microscopy (SEM) and energy dispersive X-ray analysis (EDX) the samples were placed on a carbon conductive double coated tab and sputter-coated with a thin film of gold. Microscopy was conducted in a QuantaTM 250 field-emission environmental scanning electron microscope (FEI Europe B.V., Vienna, Austria) with a Shottky field emission gun operating at 10 kV in high vacuum of 1 × 10^−6^ mbar. Differential scanning calorimetry (DSC) data of both, untreated and thermostabilised, lignins were obtained with a DSC 200F3 Maia device (NETZSCH, Graz, Austria) and a heating rate of 10 K·min^−1^. Termogravimetric analyses were carried out using a TA Instruments TGA Q5000 (Waters GmbH, UB TA Instruments, Eschborn, Germany) with a heating rate of 20 K·min^−1^. Attenuated diffuse reflection Fourier transform infrared spectroscopy (ATR-FTIR) was performed on a Helios spectrometer (Ultrafast Systems BV, Amsterdam, The Netherlands) equipped with a Tensor 27 and a diamond crystal. Spectral resolution was 4 cm^−1^. The spectra were baseline corrected. Specific electrical resistivity was measured as described in Sánchez-González *et al.* [19]. X-ray diffraction (XRD) of the samples was performed using a 5-circle X ray diffractometer (SmartLab from Rigaku Co., Ettlingen, Germany) equipped with Cu Kα radiation, a parabolic multilayer mirror in the primary beam and a secondary graphite monochromator. The samples were measured using a 2θ step of 0.02 degrees and 4 s dwell time.

Small angle X-ray scattering (SAXS) was used to obtain information about the nanostructure of the samples. Measurements were carried out with a three pinhole SMAX-3000 SAXS camera (Rigaku Co, Ettlingen, Germany) equipped with a copper target micro focus X-ray tube (MM002+ source with a wavelength of λ = 0.1541 nm). Two dimensional scattering images were recorded with a TRITON 200 multi-wire X-ray detector. The scattering images were averaged azimuthally to gain information on the scattered intensity I(*q*) in dependence on the scattering vector *q*, which is related to the scattering angle 2θ and the wavelength λ by the Bragg-equation:

$$q=\frac{4\pi }{\lambda }\times \sin\theta$$

The obtained data were background corrected and analysed with respect to the pore structure.

At high q values the scattering intensity follows the Porod law:

$$I\left(q\right)=Pq^{-n}$$

The exponent *n* is usually close to 4 for smooth surfaces and *P* the Porod constant is proportional to the pore surface. The scattering invariant *Q* is defined by

$$Q=\int_{0}^{\infty }q^{2}I\left(q\right)dq$$

and is a measure of the total scattering of the sample, related to the volumes of pores and matrix material. The ratio *P*/*Q* is proportional to the specific inner surface *S* * = *S*/*V* of the sample with a proportionality factor given by the fractions of pores Φ_1_ and the matrix material Φ_2_, with Φ_1_ = 1 − Φ_2_:

$$\frac{P}{Q}=\frac{1}{\pi \Phi_{1}\Phi_{2}}S^{*}$$

The Guinier approximation was used at low *q* to evaluate a pore radius of spherical pores *R* from the radius of gyration *R*_g_, by *R*^2^ = 5/3 $R_{g}^{2}$ with:

$$I\left(q\right)\sim I_{0}e^{-q^{2}R_{g}^{2}/3}$$

### 2.4. Preparation and Characterisation of Poly(Lactic Acid) Composites Containing Different Amounts of Lignin-Derived Carbon Microparticles

Poly(lactic acid) (PLA) was dissolved in chloroform to give a concentration of 100 g·L^−1^. Defined amounts of lignin derived carbon microparticles (0–35 wt % related to the mass of composite) were then dispersed under stirring (ultra-turrax) in 100 mL aliquots of the PLA solution. After transferring the dispersions into petri dishes, the solvent was allowed to evaporate, slowed down by filter paper put on top of the petri dishes to obtain smooth polymer films. After about 16 hours the formed films were removed from the petri dishes, folded right in the middle obtaining a double-layer of film and hot pressed at 150 °C. This procedure was repeated three-times in order to improve particle dispersion in the polymer. A final film thickness of 0.5 mm was obtained. Strips (6 mm × 60 mm) were cut and tested for tensile strength on a Zwick Roell 20 kN testing machine (Zwick GmbH & Co. KG, Ulm, Germany) equipped with a 2.5 kN load cell. Electrical resistivity of the composite films was determined according to EN ISO 3915 using the same type of strips.

## 3. Results and Discussion

### 3.1. Scanning Electron Microscopy (SEM)

**Figure 1:**
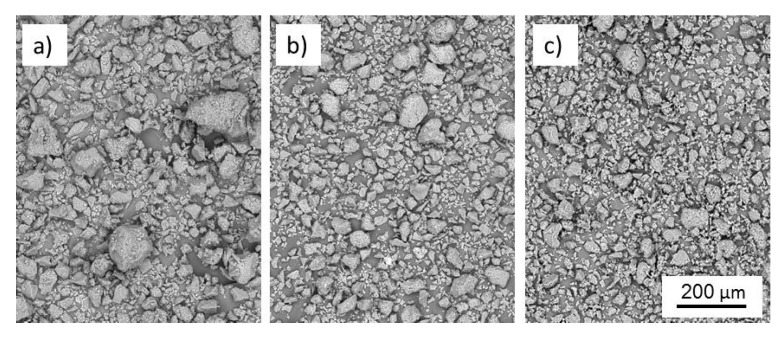
SEM micrographs of organosolv particles prior to (**a**) and after thermostabilisation (**b**) and carbonisation, respectively (**c**).

Morphological investigation of the prepared lignin-derived carbon microparticles using SEM revealed a largely irregular shape and broad size distribution for all of the three types of particles studied, *i.e.*, untreated lignin, thermostabilised lignin, and carbonised lignin (Figure 1). However, it can be seen that the particle size distribution is significantly affected by both thermostabilisation and carbonisation, as smaller particles are obtained with increasing mass loss by thermal degradation.

**Figure 2:**
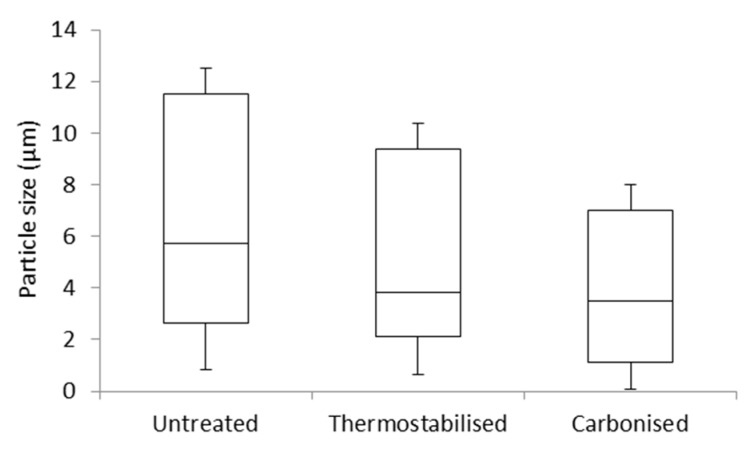
Particle size distribution of untreated, thermostabilised and carbonised organosolv lignin particles (outliers are not displayed; the box and whisker diagram is based on the minimum (lower whisker), the first quartile (bottom edge), the median (centre line), the third quartile (upper edge) and the maximum (upper whisker)).

The average particle size of the native lignin was 9.1 ± 10.3 µm, dropping to 7.0 ± 7.5 µm for the stabilised particles. The smallest average particle size was obtained for the carbonised particles (5.3 ± 6.4 µm). Box and whisker plots visualize skewed sized distributions for all types of particles in favor of smaller particles (Figure 2). A few large outliers with dimensions above 14 µm are not shown in Figure 2, and their occurrence diminishes with every treatment step.

Thermostabilisation (oxidative environment, *T* ≤ 250 °C) and carbonisation (inert gas, *T* ≤ 2000 °C) of organosolv lignin particles were accompanied by significant mass losses that accounted for 58% and 81%, respectively, of the starting material. In view of the only moderate reduction in particle size observed, the substantial mass loss measured points towards increased porosity of the particles after treatment. Compared to the literature, the reduction in particle size and the mass loss observed in the present study is less pronounced than in the case of carbonised lignosulfonate, where an overall carbon yield of only 7.3% was obtained [20].

### 3.2. Energy-Dispersive X-ray Spectroscopy (EDX)

**Figure 3:**
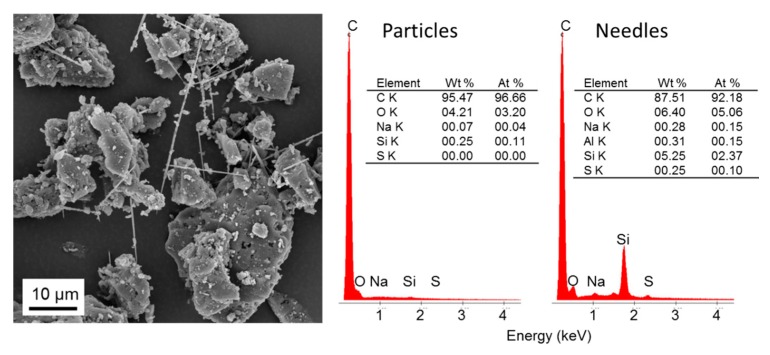
SEM image of carbonised lignin containing small amounts of needle-shape crystals (**left**) and results of EDX analyses (**right**) of both carbon particles and crystals.

The EDX results shown in Figure 3 confirm extensive carbonisation of lignin after treatment at 2000 °C, with more than 95% of sample mass converted to carbon. Besides oxygen and a trace of sodium, a small amount of silica was also detected. According to the silicon content of the parent material which was determined with 0.04 at % and the total mass yield of 19% there is a calculated total maximum of 0.2% silicon, which is in the same order of magnitude as EDX results. In good agreement with the fact that silica was detected in the carbonisation product, scanning electron microscopy revealed the presence of needle-shaped crystallites among the formed carbon particles (Figure 3). In these needles, reduced carbon content of 92.18 at % and oxygen and silicon contents of 5.06 at % and 2.37 at %, respectively, were found.

It is well known that lignin of annual plants has a high content of silicates [21], so the organosolv lignin might be produced by annual plants such as grass or bamboo. Other studies showed that standard carbon black has a characteristic O/C atomic ratio of ≤0.1, which is much lower than the measured O/C atomic ratio of our sample [22].

### 3.3. Attenuated Diffuse Reflection Fourier Transform Infrared Spectroscopy (ATR-FTIR)

**Figure 4:**
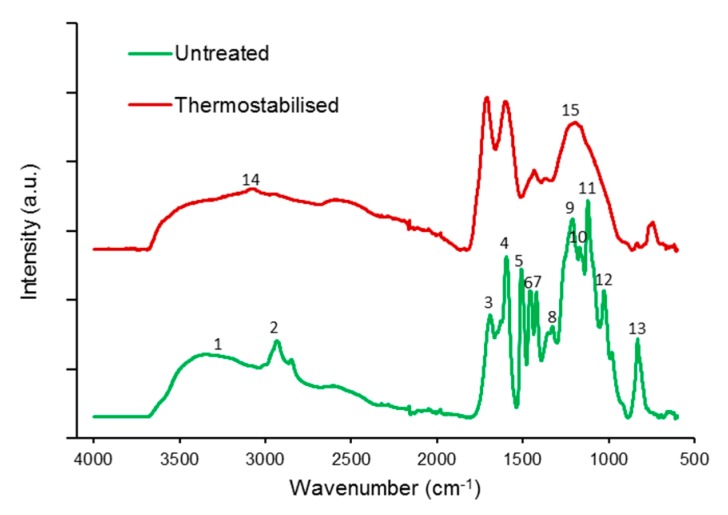
FTIR spectra of organosolv lignin particles before and after thermostabilisation.

The ATR-FTIR spectrum of unmodified organosolv lignin (Figure 4) as recorded in the vibration range of 4000–600 cm^−1^ (16 repetitions) was in good agreement with data published in Hage *et al.* [18]. Peak assignment based on the work of Norberg *et al.*, Sharma *et al.*, Braun *et al.* and Kubo *et al.* [23,24,25,26] confirmed the presence of hydroxyl groups due to phenolic or alcoholic components as shown in band (1) in the range of 3100 to 3500 cm^−1^. Band (2) at wavenumber of 2934 cm^−1^ displays aliphatic and aromatic carbons, and band (3) at wavenumber of 1690 cm^−1^ shows unconjugated groups of carbonyls. Bands (4) and (5) are “the fingerprint region” which means that they are typical for ATR-FTIR spectra of lignin and are associated with the aromatic ring modes at wavenumbers 1596 and 1510 cm^−1^, respectively. The band (6) at 1459 cm^−1^ represents C–H stretching of the aromatic skeletal, same as band (7) 1421 cm^−1^. The bands (8), (9), and (11) at the wavenumbers 1328, 1212 and 1122 cm^−1^, respectively, display syringyl and guaiacyl units. The band (10) at 1168 cm^−1^ represents conjugated esters. Finally, band (12) at 1028 cm^−1^ is due to lignin-linked hemicelluloses and band (13) at wavenumber 832 cm^−1^ is caused by aromatic C–H out of plane bending.

After thermostabilisation, the FT-IR spectra lack peaks at bands (1) and (2) indicate a reduction of phenolic or alcoholic groups, and removal and methyl groups, respectively. In parallel, band (14) at wavenumber 3073 cm^−1^, associated with aromatic hydrocarbon, increases in intensity in the thermostabilised variant. In the fingerprint region, a very significant increase is seen in band (3) associated with the stretch of unconjugated carbonyls, probably an indicator for rearrangement and radical reactions with introducing oxidised structures to the lignin. Furthermore, the number of distinct bands in the spectral region between 1500 and 700 cm^−1^ is much reduced, with a broad peak remaining at position (15) at 1199 cm^−1^. This change in spectral features shows that carbonyl groups and methoxyl were removed during autoxidation reactions. Furthermore, crosslinking reactions in the C5 position of g-lignin units occur during the thermostabilisation. In summary, it can be said that there occurred dehydration and oxidation as well as carbonyl group formation and elimination during thermostabilisation in parallel to a decomposition of aliphatic units [27,28,29,30].

The thermal stabilisation process leads to lower content of oxygen, carbon and hydrogen caused by dehydration reactions, condensation, cross linking, autoxidation and elimination reactions. Byproducts of those reactions are water, carbon monoxide and carbon dioxide which are produced via formation of aromatic, keto, anhydride and ester linkages and formation of C–C and C=C bonds [25,31,32,33,34].

### 3.4. Differential Scanning Calorimetry (DSC) and Thermogravinetric Analysis (TGA)

**Figure 5:**
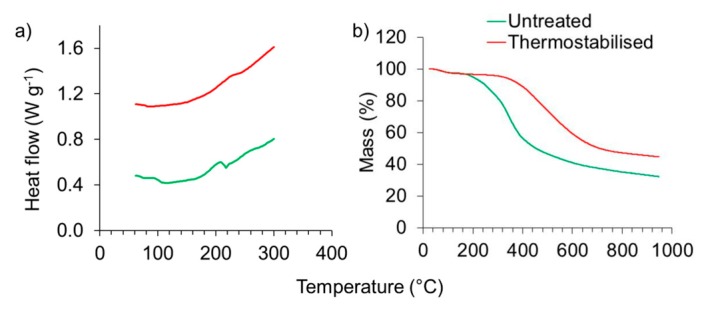
DSC (**a**) and TGA (**b**) curves of organosolv lignin particles prior to and after thermostabilisation.

Differential scanning calorimetry (DSC) and thermogravimetric analysis (TGA) were conducted to examine whether the thermostabilisation was effective in terms of eliminating thermoplasticity of the raw material. The DSC graphs of both untreated and thermostabilised lignin show an exothermal behaviour during heating from ambient temperature up to 300 °C (Figure 5a), which is more pronounced in the thermostabilised lignin compared to the untreated sample. A small but clear local minimum in the DSC curve of the untreated sample at a temperature of 230 °C indicates softening. As this local minimum vanishes entirely after thermostabilisation, this procedure is expected to be efficient in terms of preventing thermal fusing of the lignin during carbonisation.

TGA showed the mass loss, thermal and oxidation stability and the decomposition temperature of the lignin powders during heat treatment under nitrogen atmosphere (Figure 5b). There was an insignificant initial mass loss due to moisture removal [27]. The start of mass loss of the untreated lignin was detected at 170 °C. The thermogravimetry curve of the thermostabilised lignin shifted toward a higher temperature with the start of mass loss at 300 °C, which indicates an increasing thermal stability and longer temperature resistivity, longer stability and a later decomposition. The overall loss pattern was determined with 67% for the untreated lignin sample and in contrast to this the mass loss of the thermostabilised lignin was observed with 58% which approximates a total mass loss of 82% compared to the starting mass. The stagnation of the drop of the thermogravimetry curve shows that the decomposition of lignin terminates due to charring [35]. The results of TGA are in line with what was shown with DSC, the thermostabilisation was successfully conducted.

It should be mentioned here that it is known that the stabilisation process of lignin and the properties of the carbonised products largely depend on the lignin source (e.g., hardwood or softwood) because of important differences in composition and molecular weight distribution [36].

### 3.5. X-ray Powder Diffraction

**Figure 6:**
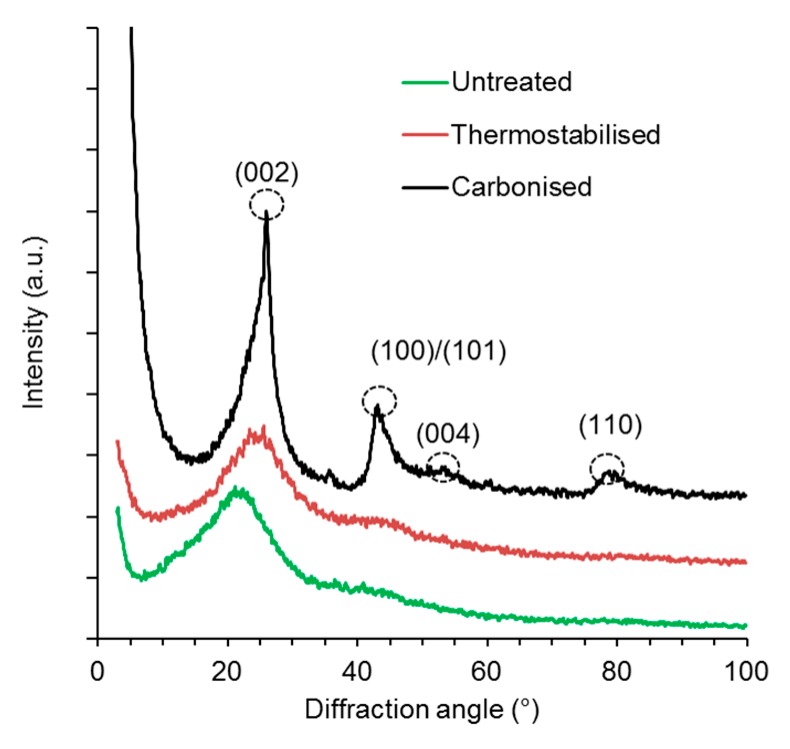
XRD patterns of organosolv lignin microparticles prior to and after thermostabilisation and carbonisation.

Structural alterations of organosolv lignin as a result of thermostabilisation and carbonisation were confirmed by X-ray diffractometry (Figure 6).

The XRD patterns of the untreated and the thermostabilised lignin powder are almost congruent and do not show well-defined peaks, which indicates a non-crystalline powder. After carbonisation, the XRD patterns show four peaks at diffraction angles of 26.7° (002), 43.2° (100)/(101), 54.7° (004) and 78.1° (110) confirming the occurrence of structural changes during carbonisation. Supramolecular rearrangement to partly graphitic structures is evident from sharpening of the (002) and (100)/(101) diffraction peaks and appearing of (004) and (110) peaks [37]. Graphite structures are formed during carbonisation by lignin dehydration, decarbonylation, decarboxylation as well as rearrangement and extension of the network of sp2 hybridised carbon formed by aromatic moieties inherent to lignin [24]. This takes place because the fundamental structure of the lignin is not affected for the most part, only side groups are cleaved and faded. Thus, graphene-like structures are built during carbonisation because of the dissociation of non-carbon atoms [6,38].

### 3.6. Small Angle X-ray Scattering

**Figure 7:**
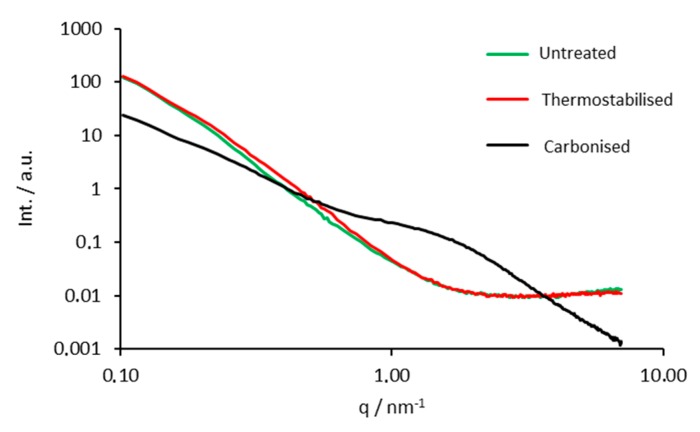
SAXS curves of the untreated, thermostabilised and carbonised organosolv lignin particles.

Small angle X-ray scattering experiments revealed a very similar morphology for the lignin particles prior to and after oxidative thermo-stabilisation, as evident from the similar decay of the scattering curves with increasing *q* values (Figure 7). Both curves can be fitted using an exponent close to four. The latter and the absence of any special feature suggest the material composed of poorly defined structures that feature very broad size distribution at the nano-scale. The existence of a slightly broader shoulder centered at about *q* = 0.5 nm^−1^ for the thermo-stabilised sample however is indicative for the development of some nano-porous structures during the oxidative activation step. In contrast, the broad peak of the scattering curve of the carbonised sample at a higher scattering vector (0.6 ≤ *q* ≤ 4) unambiguously reveals the formation of a highly nano-porous material of distinct size.

Application of the Guinier evaluation reveals a mean pore radius of *R* = (1.06 ± 0.04) nm for the carbonised lignin powder, assuming spherical pores. Also the exponent in the Porod regime is less than four, indicating a higher surface roughness. The *P*/*Q* ratio representing the total pore surface (open and closed pores) per volume increases slightly from 97 to 130 m^2^·cm^−3^ during thermostabilisation but strongly from 130 to 770 m^2^·cm^−3^ during carbonisation (*cf.* Table 1).

In conclusion, the conducted SAXS experiments confirmed that the parent lignin powder had no defined pore structure at the nanoscale. Thermostabilisation did not alter the nanostructure significantly, only a slight increase of specific surface and indication for a somewhat higher nanoporosity was found (higher scattering intensity at respective q values). After carbonisation the material shows a clear pore structure, corresponding to the appereance of a distinct shoulder in the scattering curve, attributed to a pore size of about 1 nm and an increase of the specific surface by a factor of six (770 m^−2^·cm^−3^), compared to the thermostabilised sample. The decreased exponent of the power law in the Porod regime of the scattering curve can be related to a higher surface roughness of the material.

### 3.7. Specific Electrical Resistivity

**Figure 8:**
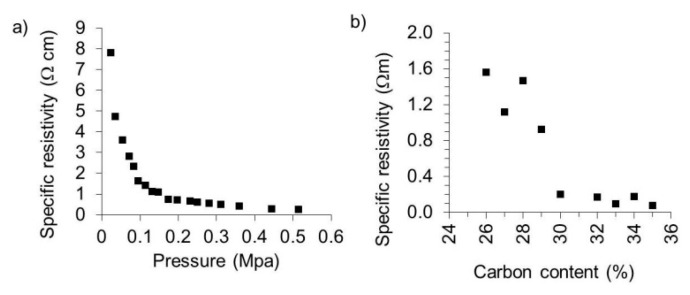
(**a**) Pressure dependence of specific resistivity for carbon particles made of organosolv lignin particles; (**b**) Effect of carbon microparticle content on specific resistivity of poly (lactic acid).

The specific electrical resistivity of the carbonised lignin particles was determined to evaluate their potential application for imparting organic polymers electro-conductivity. It can be seen in Figure 8 that the specific resistivity of carbon particles obtained in this study is in the range of 0.079 Ωm under atmospheric pressure, and 0.001 Ωm at a pressure of 0.51 MPa (Figure 8a). The resistivity diminishes with higher pressure. These findings are in good agreement with recent studies of electrical conductivity of carbon based particles derived from lignin [19,39,40,41]. It is known that heteroelements on the surface of carbonaceous materials can decrease total conductivity [39]. As mentioned, the carbon particles obtained in this study consisted of 96.66 at % of carbon and 3.20 at % of oxygen (*cf.* Figure 3). In comparison, carbon black particles obtained in a very similar way were reported to have an electrical resistivity of 0.004 Ωm under atmospheric pressure up to 0.025 Ωm at a pressure of 0.48 MPa, with a resistivity highly dependent on the extent of structural homogeneity of the prepared carbon blacks [19]. Increasing structural homogeneity generally translates into decreasing resistivity.

### 3.8. Conducting Poly(Lactic Acid) (PLA) Carbon Composites

**Figure 9:**
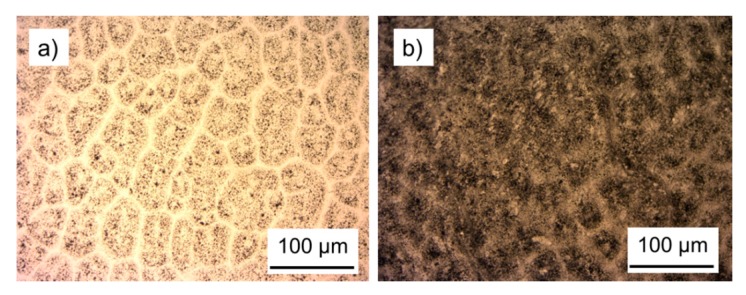
Optical micrographs of PLA films with 10% filler content showing (**a**) poor dispersion of carbon particles after casting from solution and (**b**) improved dispersion after repeated hot-pressing.

Poly(lactic acid)/carbon composites were produced by incorporating different amounts of the lignin-derived carbon powders obtained in this study into diluted solutions of PLA in chloroform and subsequent evaporation of the organic solvent. PLA was chosen as matrix polymer because this polymer is easy to handle, biodegradable and thermoplastic. It has evoked a great interest and is one of the most interesting biopolymers. There is a wide range of applications for PLA, especially for medical and biological tasks as well as packaging [42]. The obtained composite materials were found to become electrically conductive above a carbon loading of 26% (Figure 8b). Beyond this percolation threshold, conducting pathways are increasingly established within the polymer as reflected by resistivity that dropped from 1.6 Ωm (26% loading) to 0.21 Ωm when 35 wt % of carbon particles were incorporated [43]. However, compared to other studies, carbon loading to achieve electro-conductivity is still very high. Carbon black for example has been reported to impart high electrical conductivity to high-density polyethylene (HDPE) already at loading of 3 wt % [44]. In a recent study it was demonstrated that graphitised carbon black as filler in Poly(l-lactide) (PLLA) caused a decrease of resistivity from 1.6 × 10^3^ Ωm for unfilled PLLA to values lower than 1 Ωm with a concentration of carbon black of 10 wt % [45]. It is assumed that insufficiently homogeneous dispersion of particles (Figure 9) that were not surface-modified to improve compatibility with the PLA matrix may be one of the reasons for the high loadings required to reach the percolation threshold. The simple folding and hot-pressing method applied in our study clearly improved carbon dispersion in the PLA films cast from solution. However, further improvements could be achieved by proper melt-compounding by means of e.g. extrusion. It is known that the tunneling distance of electrons in carbon black-polymer systems depends on many factors and that there is no linear collective effect [9]. Liu *et al.* found that electrical resistance can be described by the tunneling conduction and percolation theory but that the electrical conductivity is dependent of the radius position [46]. In contrast, the tunneling distance of electrons in carbon nanotube polymer matrixes was found to be 1.8 nm [47]. The network of the carbon particles has to have smaller distance than this tunneling distance, otherwise there will be no electrical conductivity. The agglomeration of lignin-derived carbon powder in the PLA may be a reason for the need for high loadings of carbon powder. To reach a lower percolation threshold, the surface area can be enhanced, the structure should be higher and the volatile content should be the lowest possible [48].

### 3.9. Mechanical Properties of Poly(Lactic Acid) (PLA) Carbon Composites

**Figure 10:**
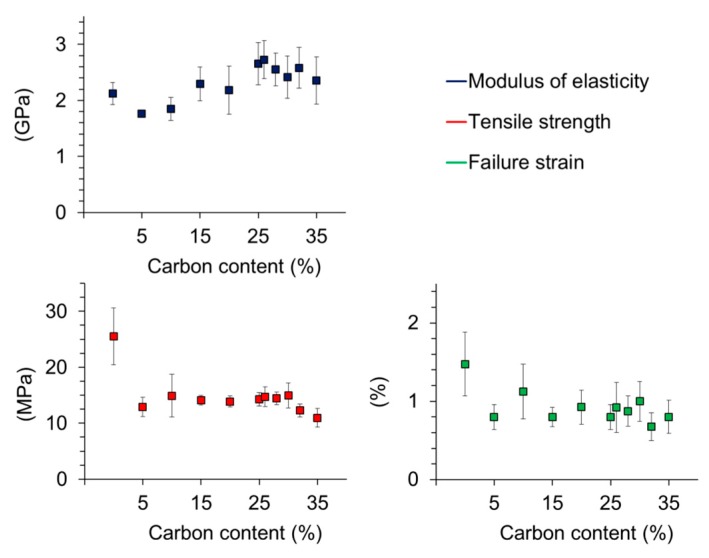
Relationship between mechanical properties and lignin-based carbon microparticle loading of poly(lactic acid) composites.

Tensile testing of PLA specimen loaded with different amounts of carbon particles revealed that both tensile strength and failure strain decreased with the addition of carbon particles to a certain extent (Figure 10). The tensile strength dropped from 25 MPa for PLA without lignin particles to 13 MPa for PLA filled with 5% carbonaceous lignin powder. For PLA filled with 5%–35% of the carbonaceous powder, the tensile strength is almost steady. Failure strain also decreased. Without carbon filling it was about 1.5%. After adding only 5% lignin derived carbon, the failure strain fell to 0.8%. Between 5% and 35% carbon loading, the failure strain was almost steady. Other studies found that the tensile strength increases with low loadings and decreases with high loadings. It was also found that the influence of carbon black particles differs a lot depending on the polymer matrix material [48]. In this case, agglomerates of the carbon particles can lead to imperfections in mechanical testing. The largest carbon particles, which were measured with 60 µm, can also lead to breakages.

In contrast to the decrease of tensile strength and failure strain, the Young modulus E seems to increase slightly at higher loadings. Overall, the effects of carbon filler on the mechanical performance of PLA are in agreement with other studies [49,50]. As outlined in a review on mechanics of particulate-filled polymers [51] an increase in composite stiffness in parallel with a reduction in tensile strength and extensibility is principally observed upon addition of particulate fillers. However, as shown in Figure 10, the PLA-carbon composites produced in the present study do not show a distinctly proportional relationship between filler content and change in mechanics, which one would expect. As with electrical conductivity of the PLA composites, it is proposed that improved filler dispersion by e.g. extrusion may lead to clearer relationships through reduced variability.

## 4. Conclusions

The results of this study are encouraging with respect to the production of conducting polymers using carbon particles derived from a specific technical lignin that is commercially available and produced by organosolv pulping of grass lignin. Even though reasonably low electrical resistivity was measured for the carbonised lignin particles, comparatively high carbon loadings were required to impart conductivity to poly(lactic acid), indicating that further research on lignin-based carbon particles is necessary.

## Figures and Tables

**Table 1:** Pore radius of the untreated, thermostabilised and carbonised lignin samples. Errors of the respective fit and calculations, respectively, are given in brackets.

Sample Name	*n*	*P*/*Q* (m^2^·cm^−3^)	*R*/(nm)
untreated	−3.85(5)	97(20)	-
thermostabilised	−3.87(2)	130(30)	-
carbonised	−3.45(2)	770(50)	1.06(4)
